# The Cumulative Effects of Polymorphisms in the DNA Mismatch Repair Genes and Tobacco Smoking in Oesophageal Cancer Risk

**DOI:** 10.1371/journal.pone.0036962

**Published:** 2012-05-18

**Authors:** Matjaz Vogelsang, Yabing Wang, Nika Veber, Lamech M. Mwapagha, M. Iqbal Parker

**Affiliations:** 1 International Centre for Genetic Engineering and Biotechnology, Cape Town Component, South Africa and Division of Medical Biochemistry, and IIDMM, University of Cape Town, Cape Town, South Africa; 2 Department for Biosynthesis and Biotransformation, National Institute of Chemistry, Ljubljana, Slovenia; IPO, - Inst Port Oncology, Portugal

## Abstract

The DNA mismatch repair (MMR) enzymes repair errors in DNA that occur during normal DNA metabolism or are induced by certain cancer-contributing exposures. We assessed the association between 10 single-nucleotide polymorphisms (SNPs) in 5 MMR genes and oesophageal cancer risk in South Africans. Prior to genotyping, SNPs were selected from the HapMap database, based on their significantly different genotypic distributions between European ancestry populations and four HapMap populations of African origin. In the Mixed Ancestry group, the *MSH3* rs26279 G/G versus A/A or A/G genotype was positively associated with cancer (OR = 2.71; 95% CI: 1.34–5.50). Similar associations were observed for *PMS1* rs5742938 (GG versus AA or AG: OR = 1.73; 95% CI: 1.07–2.79) and *MLH3* rs28756991 (AA or GA versus GG: OR = 2.07; 95% IC: 1.04–4.12). In Black individuals, however, no association between MMR polymorhisms and cancer risk was observed in individual SNP analysis. The interactions between MMR genes were evaluated using the model-based multifactor-dimensionality reduction approach, which showed a significant genetic interaction between SNPs in *MSH2, MSH3* and *PMS1* genes in Black and Mixed Ancestry subjects, respectively. The data also implies that pathogenesis of common polymorphisms in MMR genes is influenced by exposure to tobacco smoke. In conclusion, our findings suggest that common polymorphisms in MMR genes and/or their combined effects might be involved in the aetiology of oesophageal cancer.

## Introduction

According to the GLOBOCAN 2008 database (http://globocan.iarc.fr) oesophageal cancer is the 8^th^ most common cancer worldwide and the sixth most common cause of cancer death in the world, with more than 95 percent of the cases and deaths occuring in developing countries. The highest incidence rates were observed the Black population in Southern Africa and Eastern Asia, with 16.3 and 14.2 cases per 100.000 population, respectively, in contrast to Central America, Western and Central Africa where 1.4, 1.2 and 1.1 cases per 100.000 were reported, respectively. The latest report from the South African National Cancer Registry confirms this high incidence rates among Black Ancestry males and females with 8.0 and 4.5 cases per 100.000, respectively, as well as among Mixed Ancestry males and females with 10.4 and 4.4 cases per 100.000 population, respectively [1]. The main histological types of oesophageal cancer - squamous cell carcinoma (OSCC) and adenocarcinoma (OAC) - are observed in more than 95% of all oesophageal cancer cases, with OSCC being the most predominant type in Africa and China [2], [3].

Numerous alterations in certain key genes are linked with altered risks for developing oesophageal cancer [4], [5]. These genes are mainly involved in DNA maintenance and repair, alcohol, folate and carcinogen metabolism, cell cycle regulation and apoptosis. However, only a few putative genes have been consistently shown to correlate with disease susceptibility, including *ALDH2*, *CYP1A1* and *MTHFR* (reviewed in Hiyama *et al*. [5]). This suggests that there are most likely additional background genetic factors and interactions that contribute to oesophageal pathogenesis [4]. Interestingly, from over 100 genetic association studies conducted to date, only Liu *et al.* [6] study has focused on highly polymorphic genes involved in the DNA mismatch repair pathway and their role in oesophageal pathogenesis. Several genome-wide association studies (GWAS) have also been conducted in different populations, and study in European population reported associations in the DNA repair gene *HEL308* [7], [8], [9]. Moreover, MMR genes and their polymorphisms were reported to contribute to the risk of developing lung or head and neck cancer; both types of cancer share similar aetiology to oesophageal cancer [10], [11], [12], [13], [14].

Genetic or epigenetic alterations in MMR genes can completely or partially impair MMR efficiency and thus confer an increase in the accumulation of replication errors (RER) in important cancer-regulating genes, eventually leading to carcinogenesis [15]. Loss of mismatch repair activity is manifested in a microsatellite instability (MSI) phenotype. Studies investigating widespread microsatellite alterations in oesophageal cancer have detected low-level MSI (MSI-L; where at least one microsatellite locus is altered) in 16–67% of adenocarcinomas, whereas 2–60% of squamous cell carcinoma tumors were MSI-L positive, with the highest MSI frequencies observed in high-incidence populations, indicating that MMR might be involved in the pathogenesis of the oesophagus [16], [17], [18], [19], [20], [21], [22], [23], [24], [25], [26], [27], [28], [29].

These reports prompted us to investigate common variants in MMR genes and their role in susceptibility to oesophageal squamous cell carcinoma in a high-risk population. We performed a case-control study in two distinct ethnic groups of South Africans, where we examined potential associations between 10 polymorphisms in 5 MMR genes (*MLH1*, *MLH3*, *PMS1*, *MSH2* and *MSH3*) and oesophageal cancer. Moreover, SNP-SNP interactions, as well as SNP-environment interactions were investigated to further examine involvement of MMR system in OC.

## Results

### Case-control Single-SNP Analysis

Characteristics of the two groups, Black and Mixed Ancestry are provided in Table 1. In Black Africans, cases and controls were similar in terms of age (*P* = 0.109) and family history of cancer (*P* = 0.920). In the Mixed Ancestry group, cases compared with controls were more likely to be males, smoke and drink alcohol (*P*<0.0001 for all) and there was no significant difference in the age distribution (*P* = 0.472) between cancer cases and cancer-free controls (Table 1). A combination of smoking and drinking habits increased the risk for oesophageal cancer 5.46-fold in Black and 19.06-fold in Mixed Ancestry populations (*P*<0.0001 for each population; Table 2).

**Table 1:** Characteristics of study subjects.

Characteristics	Black Ancestry group	Mixed Ancestry group			
	Controls (%)	Cases (%)	Controls (%)	Cases (%)	
Sample size		344	345	266	205
Histology	OSCC		326 (94.5)		182 (88.8)
	OAC		19 (5.5)		23 (11.2)
Age	Mean (SD)	56.1 (16.2)	59.8 (11.3)	56.8 (16.5)	60.7 (10.2)
Gender	Female	224 (65.1)	179 (51.9)	184 (69.2)	69 (33.7)
	Male	120 (34.9)	166(48.1)	82 (30.8)	136 (66.3)
Smoking status	No	227 (66.0)	136 (39.4)	104 (39.1)	16 (7.8)
	Yes	117 (34.0)	209 (60.6)	162 (60.9)	189 (92.2)
Alcohol intake	No	252 (73.3)	185 (53.6)	228 (85.7)	87 (42.4)
	Yes	92 (26.7)	160 (46.4)	38 (14.3)	118 (57.6)
Place of birth	Eastern Cape	159 (46.2)	284 (82.3)	14 (5.3)	18 (8.8)
	Western Cape	154 (44.8)	35 (10.2)	220 (82.7)	170 (82.9)
	Other	31 (9.0)	26 (7.5)	32 (12.0)	17 (8.3)
Family history of Cancer	Yes	60 (17.4)	55 (15.9)	85 (32.0)	68 (33.2)
	No	284 (82.6)	290 (84.1)	181 (68.0)	137 (66.8)

OSCC, oesophageal Squamous cell carcinoma; OAC, oesophageal adenocarcinoma.

**Table 2:** Effects of smoking and drinking on oesophageal cancer risk.

	Black Ancestry group	Mixed Ancestry group						
	Case	Control	AOR (95% CI)	*P-*value	Case	Control	AOR (95% CI)	*P-*value
Tobacco smoking/alcohol consumtion								
*No/No*	118	206	1.00 (reference)		13	100	1.00 (reference)	
*Yes/No*	67	46	**3.15 (1.85–5.36)**	**2.30×10^−5^**	74	128	**3.76 (1.92−7.35)**	**1.09×10^−4^**
*No/Yes*	18	21	**2.32 (1.08−4.98)**	**0.031**	3	4	4.42(0.83**−**23.60)	0.082
*Yes/Yes*	142	71	**5.46 (3.28−9.10)**	**7.3×10^−11^**	115	34	**19.06 (8.96−40.55)**	**1.9×10^−14^**

Genotype and minor allele frequencies for SNPs are shown in Table S1. For polymorphism rs26279 (*MSH3*), the minor G allele occurred with a frequency of 32% in cases vs. 38% in controls in the Mixed Ancestry group (*P* = 0.044). The G allele of polymorphism rs5742938 (*PMS1*) had a frequency of 48% in cases and 57% in controls (*P* = 0.007) in the Mixed Ancestry group. The minor A allele of rs28756991 (*MLH3*) polymorphism occurred in 4% Mixed Ancestry controls vs. 9% in Mixed Ancestry cases (*P* = 0.001). No difference was observed among Black cases and controls for analysed polymorphisms. Allelic distributions in Black controls were in good agreement with those in the LWK or YRI HapMap populations. All polymorphisms were found to be in Hardy-Weinberg equilibrium (*P*>0.05) when examining Black and Mixed Ancestry controls, separately. For ten SNPs under study, more than 99% of samples were successfully genotyped. We used logistic regression analysis to examine potential associations between polymorphisms in MMR genes and oesophageal pathogenesis before and after adjusting for age, gender, place of birth, lifestyle habits and familial history of cancer. Adjusted odds ratios are represented in Table 3. Dominant and recessive models for minor alleles were considered for each SNP.

**Table 3:** Individual SNP effects on oesophageal cancer risk in two ethnic groups of South African population.

SNP	Black Ancestry group	Mixed Ancestry group			
	Genetic model	AOR (95% CI)	*P*-value	AOR (95% CI)	*P*-value
rs17217772, Asn127Ser	GG vs AA/AG	1.73(0.13**−**23.7)	0.681	ND	
	GG/AG vs AA	0.99(0.58**−**1.69)	0.976	1.48(0.59**−**3.73)	0.402
rs10188090, c.2635**−**765G>A	GG vs AA/AG	1.13(0.24**−**5.44)	0.879	1.33(0.65**−**2.69)	0.434
	GG/AG vs AA	1.02(0.65**−**1.60)	0.927	0.83(0.56**−**1.34)	0.510
rs3771280, c.1510+118T>C	TT vs CC/CT	1.99(0.47**−**8.39)	0.350	0.94(0.51**−**1.74)	0.839
	TT/CT vs CC	1.07(0.71**−**1.63)	0.735	0.80(0.51**−**1.25)	0.325
rs26279, Ala1045Thr	GG vs AA/AG	0.82(0.52**−**1.30)	0.399	**2.71(1.34−5.50)**	**5.71×10^−3^**
	GG/AG vs AA	0.93(0.64**−**1.33)	0.674	1.32(0.83**−**2.08)	0.238
rs1428030, c.1341-12568A>G	GG vs AA/AG	0.90(0.48**−**1.69)	0.747	1.56(0.57**−**4.31)	0.390
	GG/AG vs AA	1.36(0.96**−**1.92)	0.086	1.13(0.72**−**1.77)	0.605
rs1805355, Pro231Pro	AA vs GG/GA	0.61(0.32**−**1.15)	0.128	1.25(0.47**−**3.36)	0.656
	AA/GA vs GG	1.14(0.81**−**1.61)	0.464	1.02(0.65**−**1.59)	0.945
rs5742938, c.-21+639G>A	AA vs GG/GA	2.29(0.67**−**7.79)	0.182		
	AA/GA vs GG	1.04(0.72**−**1.51)	0.843		
	(GG vs AA/AG)^a^			**1.73(1.07−2.79)**	**0.027**
	(GG/AG vs AA)^a^			1.32(0.79**−**2.19)	0.281
rs13404927, c.699+3331G>A	AA vs GG/GA	0.56(0.23**−**1.38)	0.210	0.97(0.24**−**3.92)	0.969
	AA/GA vs GG	1.04(0.72**−**1.50)	0.848	1.17(0.69**−**1.95)	0.561
rs13320360, c.546-191T>C	CC vs TT/CT	0.54(0.17**−**1.73)	0.299	7.16(0.49**−**104.49)	0.150
	CC/CT vs TT	0.86(0.59**−**1.26)	0.439	1.12(0.56**−**2.18)	0.772
rs28756991, Arg797His	AA vs GG/GA	0.14(0.02**−**1.25)	0.078	ND	
	AA/GA vs GG	0.86(0.57**−**1.31)	0.488	**2.07(1.04−4.12)**	**0.038**

a Minor alleles are different between the two ethnic groups, hence genetic model indicated in brackets was investigated in Mixed Ancestry group. Significant associations are printed in bold.

AOR, odds ratio adjusted for age, gender,smoking status, alcohol intake, place of birth and family history of cancer; CI, confidence interval; ND, not determined (zero genotypes were found in one genotype group).

In the Mixed-ancestry group, the *MSH3* rs26279G/G versus A/A or A/G genotype was positively associated with cancer (adjusted OR = 2.71; *P* = 5.71×10**^−^** ^3^). Similar associations were observed for *PMS1* rs5742938 (GG versus AA or AG: ajdusted OR = 1.73; *P* = 0.027) and *MLH3* rs28756991 (AA or GA versus GG: adjusted OR = 2.07; *P* = 0.038). We found that all three associations remained significant after correcting the *P*-values for multiple testing, using the Benjamini-Hochberg method (rs26279: *P*_corrected_ = 0.027; rs5742938: *P*_corrected_ = 0.036; and rs28756991: *P*_corrected_ = 0.027; [see Materials and Methods for details]). In Black South Africans, we observed a marginal association (*P* = 0.086) for *MSH3* rs1428030 polymophism (GG or AG versus AA) with a 1.36-fold increase in cancer risk after adjusting for other confounders. There was also evidence implying a reduced cancer risk, with marginal significance, for *MLH3* rs2875991 ‘A’ allele under recessive genetic model (adjusted OR: 0.14; *P* = 0.078). However, after correcting for multiple tests, significance for both associations was lost. In addition, single-SNP associations were also investigated only among squamous cell carcinoma cases, excluding adenocarcinomas, however anaysis did not provide additional or more significant results (Table S2).

### Haplotype Analysis

Haplotype analysis was performed to further evaluate the role of MMR genes in cancer aetiology. As shown in Table 4, three SNPs in *MSH3* (rs1805355, rs1428030 and rs26279) and two SNPs in *PMS1* (rs572938 and rs13404927) were used to generate haplotypes. The frequency of A_rs1805355_ **−**G_rs1428030_ **−**G_rs26279_ haplotype of *MSH3* was found to be significantly higher in black controls (6.8%) than in black cancer cases (3.6%). However, the observed inverse association was only marginally significant after correcting for multiple tests (*P*_1000_ = 0.049). In the Mixed Ancestry group, G_rs5742938_ **−**G_rs13404927_ haplotype of *PMS1* was associated with 1.61**−**(95% CI: 1.22**−**2.13) increase in OC risk, compared to the reference A_ rs5742938_ **−**G_ rs13404927_ haplotype and remained significant after correcting for multiple tests (*P*_1000_ = 0.011). Observed *PMS1* haplotype effect is entirely due to the association of the *PMS1* G_rs5742938_ allele observed in the single SNP analysis, and no increase in significance is achieved by inclusion of the variant rs13404927. There was no association between the three-marker haplotype of *MSH2* (rs17217772, rs3771280 and rs10188090) and OC risk in either of the two ethnic groups (data not shown).

**Table 4:** Estimated frequencies of haplotypes for the *MSH3* and *PMS1* genes.

Haplotype	Black Ancestrygroup	Mixed Ancestry group							
	Control (%)	Cases (%)	OR^a^ (95% CI)	*P-*value	Control (%)	Cases (%)	OR^a^ (95% CI)	*P-*value	
*MSH3* rs1805355 : rs1428030 : rs26279	G A G	242 (35.1)	240 (34.7)	1.00 (reference)		127 (23.9)	114 (27.8)	1.30(0.95–1.78)	0.109
	G A A	241 (35.0)	234 (33.9)	0.98(0.76–1.26)	0.897	278 (52.3)	192 (46.8)	1.00 (reference)	
	A G A	129 (18.8)	157 (22.7)	1.23(0.92–1.65)	0.179	58 (11.0)	52 (12.8)	1.30(0.86–1.97)	0.239
	A G G	47 (6.8)	25 (3.6)	**0.54(0.32–0.90)**	**0.022**	41 (7.7)	37 (9.0)	1.31(0.81–2.11)	0.321
					0.049^b^				
*PMS1* rs5742938 : rs13404927	A G	91 (13.2)	110 (16.0)	1.22(0.90–1.66)	0.195	266 (50.0)	158 (38.4)	1.00 (reference)	
	G G	463 (67.3)	457 (66.3)	1.00 (reference)		203 (38.1)	194 (47.3)	**1.61(1.22–2.13)**	**9.0×10^−4^**
	G A	119 (17.3)	110 (16.0)	0.94(0.70–1.25)	0.655	53 (10.0)	40 (9.8)	1.27(0.81–2.00)	0.346
									0.011^b^

Only haplotypes with a frequency greater than 3.0% are listed.

a ORs were obtained with X^2^ test.

b *P-*value was obtained with 1000 permutations test. OR, unadjusted odds ratio; CI, confidence interval.

### Gene-gene Interaction Analysis

Possible cumulative effects of the SNPs were evaluated with MB-MDR approach (see Material and Methods), as it is well known that MMR enzymes function as heterodimers. Two, three and four-order interaction models were considered and the results are shown in Table 5. Data revealed best genetic interaction for SNPs in *MSH2* gene (rs3771280), *MSH3* gene (rs1428030) and *PMS1* gene (rs13404927 and rs5742938), which was strongly associated with increased risk of oesophageal cancer in Black subjects. The frequency of the four-locus genotype CC_rs3771280_/AG_rs1428030_/GG_rs13404927_/GG_5742938_ was significantly higher in cases (18.6%) compared to controls (9.3%). In the Mixed Ancestry group, three significant multigene interactions were predicted. A three-order interaction *MSH2* (rs3771280) * *PMS1* (rs13404927) * *MSH3* (rs26279) and a four-order interaction, which included the rs13320360 polymorphism in *MLH1* gene, the rs10188090 polymorphism in *MSH2* gene and the rs13404927 and rs5742938 polymorphisms in *PMS1* gene, were the most significant and were hence regarded as the best models. The multi-locus genotype CT_rs3771280_ **−**GG_rs13404927_ **−**AG_rs26279_ was strongly associated with reduced risk for cancer (*P* = 0.0028), whereas the genotype TT/AA/GG/GG from interaction *MLH1* (rs13320360) *MSH2 (rs10188090) *PMS1 (rs13404927) *PMS1 (rs5742938) was more than 2-fold higher in cancer patients than in healthy individuals (Table 5). All three aforementioned interactions, remained significant after 1000 random permutations test.

**Table 5:** Gene-gene interactions affect oesophageal cancer risk.

Order^a^	Best multigene interaction model	Cases (%)	Controls (%)	*P-*value	Risk^b^			
*Black subjects*								
2	*MSH3*(rs1428030) *PMS1(rs5742938)							
	**AA**	**GG**			**28.9**	**38.4**	**0.00993**	**Low Risk**
	AG	GG			32.2	25.6	0.05324	High Risk
	P_1000_ = 0.089							
3	*MSH2*(rs3771280) * *MSH3*(rs1428030) *PMS1(rs5742938)							
	CC	AA	GG		23.5	31.7	0.013038	Low Risk
	**CC**	**AG**	**GG**		**26.4**	**16.9**	**0.003077**	**High Risk**
	P_1000_ = 0.067							
4	*MSH2*(rs3771280) * *MSH3*(rs1428030) * *PMS1*(rs13404927) *PMS1(rs5742938)							
	CC	AA	GG	GG	13.0	22.4	0.0010468	Low Risk
	**CC**	**AG**	**GG**	**GG**	**18.6**	**9.3**	**0.0007318**	**High Risk**
	P_1000_ = 0.049							
*Mixed-ancestry* *subjects*								
2	*MLH3*(rs28756991) *MSH2(rs17217772)							
	AG	AA			14.2	7.1	0.015846	High Risk
	**GG**	**AA**			**77.1**	**86.5**	**0.003429**	**Low Risk**
	P_1000_ = 0.002							
3	*MSH2*(rs3771280) * *PMS1*(rs13404927) * *MSH3*(rs26279)							
	CC	AG	AG		5.4	1.5	0.025807	High Risk
	CT	AG	AG		5.4	1.5	0.079535	High Risk
	**CT**	**GG**	**AG**		**8.8**	**20.7**	**0.002852**	**Low Risk**
	CC	GG	GG		5.4	1.5	0.025807	High Risk
	P_1000_ = 0.013							
4	*MLH1*(rs13320360) *MSH2(rs10188090) *PMS1(rs13404927) *PMS1(rs5742938)							
	TT	AG	GG	AA	6.3	12.8	0.02427	Low Risk
	TT	AA	GG	AG	6.8	12.8	0.03856	Low Risk
	**TT**	**AA**	**GG**	**GG**	**15.1**	**6.4**	**0.00233**	**High Risk**
	P_1000_ = 0.049							

NOTE: Individuals with multi-locus genotype of interest are compared against the rest of the individuals, which are considered as a reference group in each logistic regression analysis. Low Risk, deceased risk for malignancy; High Risk, increased risk for malignancy.

a Number of SNPs considered. Genotypes with most significant effects for each interaction (i.e. lowest *P-*value) are printed in bold. Statistical significance for each interaction was further determined by 1000 permutations test (P_1000_).

### Gene-environment Interactions

To further investigate the role of MMR polymorphisms in relation to environmental factors, individuals were stratified for tobacco smoking habits. Three polymorphisms, that showed association with oesophageal cancer risk in a single-SNP analysis (see Table 3) were investigated in the stratified analysis based on smoking. In the Mixed Ancestry group, polymorphisms *MSH3* rs26279 and *MLH3* rs28756991 remained associated with the disease in smokers (*P*_rs26279_ = 0.004, and *P*_rs28756991_ = 0.011) in contrast to non-smokers, where no significant associations were observed (Table 6). In addition, three most significant gene-gene interactions were investigate after stratifying both populations for tobacco smoke exposure. Association of the four-locus genotype CC_rs3771280_/AG_rs1428030_/GG_rs13404927_/GG_5742938,_ identified in Black subjects, was associated with OC in tobacco smokers (*P* = 0.007), whereas the significance of the association of the TT_rs13320360_/AA_rs10188090_/GG_rs13404927_/GG_rs5742938_ genotype with OC in Mixed Ancestry subjects was lost (*P* = 0.054). The interaction *MSH2* (rs3771280) * *PMS1* (rs13404927) * *MSH3* (rs26279) was only significant in smokers (*P* = 0.004) in the Mixed Ancestry group (Table 6).

**Table 6:** Cumulative effects of genetic variation and tobacco smoking habits on oesophageal cancer risk.

Genetic variation	*Non smoking subjects*	*Smoking subjects*			
	Genotype	Case/Control	AOR (95% CI)	Case/Control	AOR (95% CI)
*Single SNP*					
a rs26279	AA/AG	14/95	1.00	157/148	1.00
	GG	1/8	0.78 (0.08–7.76)	32/12	**3.14 (1.43–6.92)**
a rs5742938	AA/AG	10/79	1.00	115/120	1.00
	GG	5/25	3.15 (0.78–12.74)	73/42	**1.66 (0.99–2.78)**
a rs28756991	GG	16/94	1.00	155/150	1.00
	AA/GA	0/8	ND	34/12	**2.69 (1.25–5.79)**
*Multi-order interaction*					
b (rs3771280) *(rs1428030) *(rs13404927) *(rs5742938)	Other genotypes	115/202	1.00	166/110	1.00
	CC/AG/GG/GG	21/25	1.39 (0.69–2.75)	43/7	**3.37 (1.38–8.26)**
a (rs13320360) *(rs10188090) *(rs13404927) *(rs5742938)	Other genotypes	15/100	1.00	159/149	1.00
	TT/AA/GG/GG	1/14	3.85 (0.24–61.83)	30/13	**2.12 (0.99–4.57)**
a (rs3771280) *(rs13404927) *(rs26279)	Other genotypes	16/93	1.00	171/123	1.00
	CT/GG/AG	0/11	ND	18/39	**0.36 (0.18–0.73)**

a SNP or interaction was analysed in Mixed Ancestry subjects.

b SNP or interaction was analysed in Black Ancestry subjects.

AOR, odds ratios adjusted for age, gender, place of birth, alcohol consumtion and family history of cancer; CI, confidence interval; ND, not determined (zero genotypes were found in one genotype group); Significant and border significant AORs are printed in bold.

### Functional Analysis

**Figure 1:**
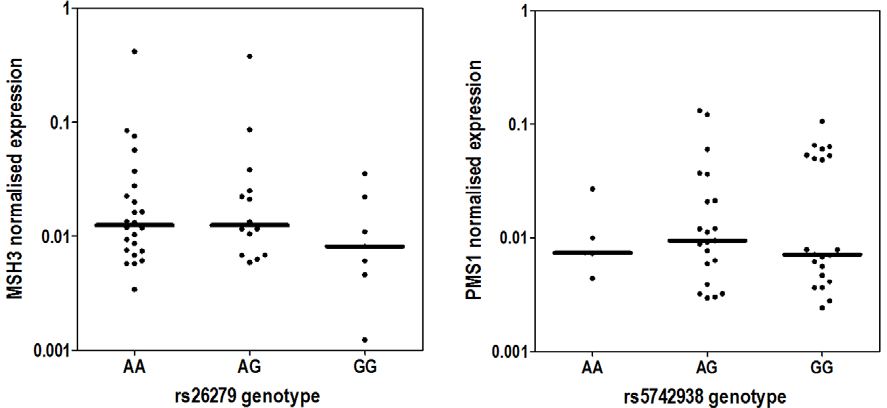
Correlations of rs26279 and rs5742938 genotypes with *MSH3* and *PMS1* mRNA expression levels. Expression levels (bars show group medians) were determined in normal tissue samples from Mixed Ancestry OSCC patients. All values are normalized to GAPDH expression. Kurskal-Wallis test was used to evaluate differences in expression levels between groups. Median values for rs26279 genotypes are: 0.012 (AA), 0.012 (AG) and 0.0081 (GG). Median values for rs5742938 genotypes are: 0.0074 (AA), 0.0094 (AG) and 0.0071 (GG).

To assess functional nature of OC-associated SNPs, that were identified in this study, *MSH3* and *PMS1* mRNA levels were examined in normal oesophageal biopsies from 47 OSCC patients in correlation with rs26279 and rs5742938 genotypes, respectively. No significant effects of the rs26279 and rs5742938 genotypes on *MSH3* and *PMS1* expression levels, respectively, were observed (*P*_rs26279_ = 0.340 and *P*_rs5742938_ = 0.954) (Fig. 1). In addition, functional and structural effects of amino acid substitutions Ala1045Thr (rs26279) in MSH3 and Arg797His (rs28756991) in MLH3 were predicted using bioinformatic algorithms SIFT, PolyPhen, and Align-GVGD. Bioinformatic tools predict whether amino acid change will have neutral or damaging impact of the protein, based on multiple alignment information and biophysical characteristics of amino acids. Evolutionary sequence conservations were prepared from 26 MSH3 and 19 MLH3 protein sequences from different species and served as an input for all algorithms. *In silico* algorithm Align-GVGD predicted neutral effect for variant Arg797His (rs28756991), whereas SIFT and PolyPhen predicted it to have damaging impact on the proteins. All three computational approaches were consistent in predicting neutral functional nature of amino acid change Ala1045Thr (Table S3).

## Discussion

Carcinogenesis is a multistep process involving genetic and environmental risk factors. Common polymorphisms in many genes, including those involved in DNA repair, have been shown to predispose individuals to the disease. Genetic alterations in microsatellite regions, a hallmark of a defective DNA mismatch repair system, have been reported in oesophageal cancers. Despite this, common polymorphisms in MMR genes have rarely been studied in relation to OSCC susceptibility.

To estimate OSCC risk conferred by common polymorphisms in MMR genes, we analysed 10 SNPs within 5 genes of the MMR pathway in high incidence populations in South Africa. In light of the common disease/common variant (CD/CV) hypothesis, SNPs were selected on the basis of their different genotypic distributions between African and non-African populations. Recent studies also indicate that interplay between multiple polymorphisms plays a key role in carcinogenesis; we therefore analysed SNP-SNP as well as SNP-environment interactions in association with OC.

In this study, we identified three common polymorphisms that were associated with OSCC in Mixed Ancestry individuals. Firstly, the GG-genotype of polymorphism *MSH3* rs26279 was positively associated with the disease. Polymorphism *MSH3* rs26279 has been examined before, however with partially conflicting results across studies. Conde *et al*. [30] observed no association with breast cancer risk at the individual-SNP level in Caucasian females. However, gene-gene interaction analysis in the same study showed that multi-locus genotype AA/TC in *MSH3* rs26279 *M*SH6* rs1042821 interaction was associated with a decreased risk for tumorigenesis, suggesting that rs26279 changes affinity of MSH3 protein to heterodimerize with MSH6. Furthermore, Liu *et al.* [6] found no association between rs26279 and oesophageal adenocarcinoma in the Caucasian population, whereas a study by Hirata *et al*. [31] reports that the GG or AG genotypes of *MSH3* rs26279 polymorphism might be a risk factor for sporadic prostate cancer. Furthermore, down-regulation of MSH3 was found to induce a MSI-L phenotype in sporadic colorectal cancer (reviewed by Boland and Goel, 2010 [32]). It is possible that a similar mechanism is responsible for frequently observed MSI-L phenotype in OSCC cases [23]. In our effort to assess functional nature of identified SNP we performed expression analysis of *MSH3* in biopsy samples from patients and did not detect any correlation between rs26279 genotypes and expression levels of *MSH3* gene. Moreover, *in silico* algorithms predicted neutral effect on the proteins’ function and structure.

Secondly, we observed that a homozygous genotype for the G-allele in the *PMS1* rs5742938 polymorphism was associated with cancer in Mixed-ancestry South Africans. This finding was further confirmed by haplotype analysis in Mixed-ancestry subjects, where haplotype G_rs5742938_ **−**G_rs13404927_ of *PMS1* increased the risk for cancer. The intronic change c.-21+639G>A (rs5742938) has not been identified in association with any type of cancer before; however, it was predicted by an UTRScan computational algorithm as functionally non-significant [33]. According to our biopsy expression analysis, genotypes of rs5742938 do not affect the *PMS1* mRNA expression levels. In the current literature the role of MLH1-PMS1 complexes in mismatch repair remains enigmatic.

Lastly, having one or two copies of an A-allele in *MLH3* rs28756991 polymorphism was associated with increased risk for developing OSCC. This finding was also supported by i*n silico* SIFT and PolyPhen algorithms, which predicted that aminoacid change Arg797His (rs28756991) has potentially damaging impact on the structure and function of MLH3 protein. This is the first study reporting on *MLH3* rs28756991 polymorphism (Arg797His) and its relation to cancer risk; however, other functional polymorphisms in the *MLH3* gene have previously been shown to confer cancer susceptibility. Michiels *et al*. [34] have shown that SNP *MLH3* rs175080 (Leu844Pro) was associated with an increased risk for lung cancer in European Caucasians. A similar finding was reported by Conde *et al*. [30] where interaction between *MLH3* rs175080 and *MSH4* rs5745325 was associated with increased risk for breast cancer in a Portuguese population. Taken together, these reports support our finding that *MLH3* could indeed be involved in the development of various types of sporadic cancers, including OSCC. MLH3 is the third protein that binds to MLH1, a key player in MMR apparatus, hence inefficient assembly of MLH1–MLH3 complex could lead to low penetrating oncogenic events.

In addition, significance for all three associations in the Mixed-ancestry group persisted after correction for multiple tests and the powers to detect the observed effect sizes were 90.47% (*MSH3* rs26279), 84.88% (*PMS1* rs5742938) and 86.71% (*MLH3* rs28756991).

We failed to confirm similar associations in Black South Africans at the single SNP level. The reason for lack of significant associations in these individuals probably lies in different linkage disequilibrium (LD) patterns among the two ethnic groups, rather than different aetiologies of OSCC. African populations are most probably the oldest populations in the world, since Africa is believed to be the continent of origin for modern humans. In older populations, the sizes of LD blocks are generally smaller, due to more recombination events [35], [36]. Hence, we can speculate that the marker alleles, which were identified in the Mixed Ancestry group (this is a young population arising from admixture of non-Africans with indigenous African populations in the 17th century), are in LD with the disease-causing alleles of *MSH3*, *PMS1* and *MLH3* genes, whereas in Black Ancestry subjects (i.e. representatives of an old population in Africa) the investigated alleles might not be in LD with the disease alleles. However, association studies in other ethnic groups are needed in support of this notion. Furthermore, populations of African origin present an opportunity to identify the true disease alleles, which are responsible for the disease phenotype, by fine-mapping of the genetic regions that are identified as disease-associated in non-African populations [37].

To further investigate the involvement of the MMR mechanism in OSCC development, we explored possible gene-gene interactions by MB-MDR, a dimension reduction method proposed by Calle *et al*. [38]. In both groups, interaction analysis yielded several statistically significant interactions. In Black individuals, a potential four-order interaction *MSH2* (rs3771280) * *MSH3* (rs1428030) * *PMS1* (rs13404927) *PMS1 (rs5742938) demonstrated strong association with increased risk for malignancy, whereas interaction *MSH2* (rs3771280) * *PMS1* (rs13404927) * *MSH3* (rs26279) significantly decreased the risk for cancer in Mixed Ancestry subjects (Table 5). Interestingly, results are consistent between the two groups, since most significantly disease-associated interactions were found between SNPs which are situated in *MSH2*, *MSH3* and *PMS1* genes. Based on our data and the knowledge that MMR activity is achieved by protein heterodimers, one could argue that SNPs, affecting functionality of the ternary complex between heterodimers MSH2–MSH3 and MLH1-PMS1, are an important event in OSCC development. We also believe that there could be many genetic interactions that affect assembly and functionality of other MMR-heterodimers and therefore these need to be identified. *MLH3* (rs28756991) *MSH2 (rs17217772) interaction was found to reduce the risk for OSCC in Mixed Ancestry population (Table 5). Moreover, similar gene-gene interactions of MMR genes have also been reported by Conde *et al.* [30] in association with breast cancer susceptibility.

Our data also implies that pathogenesis of common polymorphisms in MMR genes are influenced by environmental exposures, especially tobacco smoking. Similar findings have been reported before in other cancer types, that share common aetiology to oesophageal cancer. Hirao et al. [11] have shown association between lung cancer and loss of heterozygosity (LOH) at *MLH1* locus, with higher prevalence of LOH in smoking patients. Several studies also suggest that the *MLH1* rs1800734 polymorphism and tobacco smoke exposure have a role in tumorigenesis of lung cancer [12], [13]. In our study, polymorphisms rs26279 (*MSH3*) and rs2875661 (*MLH3*) appear to be involved in smoking-related cancer, as they were only pathogenic among smoking individuals in contrast to non-smoking, where no significant association was observed (Table 6). The powers to detect the observed effect sizes in tobacco-smoking cases of Mixed Ancestry group remained sufficient for *MSH3* rs26279 (97.53%) and *MLH3* rs28756991 (85.05%), whereas borderline association of *PMS1* rs5742938 with cancer was underpowered at 64.06%. We are aware that only as few as 16 non-smoking Mixed Ancestry cases were present in the study, which considerably reduced the power to detect the observed effect sizes in non-smokers. Therefore, to further support the results obtained from Mixed Ancestry group, stratification analysis was performed on the Black Ancestry group, where more smoking and non-smoking individuals were enrolled. The four-order interaction identified by MB-MDR was strongly associated with cancer in smoking Black Ancestry individuals, in contrast to non-smoking individuals of the same ethnic group, where no association was observed (Table 6). This trend suggests that defective MMR proteins - their activity may be compromised by polymorphisms in MMR genes - might be inefficient in repairing increased amounts of smoking-induced DNA adducts and/or signalling for apoptosis in such DNA error events. Our results support the data obtained by Dodd *et al*. [39], where it was reported that genes involved in metabolism of nitrosamines and DNA repair processes, including MMR, are dysregulated in nasopharyngeal carcinoma (NPC). These authors proposed an interplay between exogenous exposure to sources of nitrosamines (such as dietary, tobacco smoke and other), and the ability to efficiently metabolize nitrosamines or repair DNA damage induced by reactive byproducts of nitrosamine metabolism in the aetiology of NPC. Nitrosamine 4- (methylnitrosamino)-1-(3-pyridyl)-1-butanone (NNK) is a potent carcinogen contained in the cigarette smoke and was shown to induce cellular DNA damage [40], [41], [42]. A study by Hou *et al*. [43] reported that Bcl2 enhances the frequency of NNK-induced mutations by down-regulating MMR efficiency via disruption of the MSH2-MSH6 complex. Despite this, observed gene-environment interactions associated with OSCC still warrant confirmation in a larger independent study.

In addition, we confirmed from additive (in Black group) to synergistic (in Mixed Ancestry group) risk effect of tobacco smoke and alcohol combination on carcinogenesis as previously reported by several other studies [44], [45], [46].

In conclusion, this study provides evidence that common polymorphisms in MMR genes, are indeed involved in the aetiology of OSCC. Cumulative effects of MMR-polymorphisms were further shown to strongly contribute to cancer development in both ethnic groups. In fact, our results imply that combined effects of common polymorphisms in MMR genes might alter susceptibility to OSCC by modulating the effect of exposure to first-hand tobacco smoke.

## Materials and Methods

### Study Subjects

The study population has been described elsewhere [47], [48], [49]. Briefly, a total of 1239 individuals were recruited from Black and Mixed Ancestry population of South Africa. Black individuals (n = 689) were Xhosa-speaking South Africans (Xhosa-speakers originated from the Bantu-speakers in Southern Africa), mostly born in regions of the Eastern and Western Cape. Subjects resulting from marriages between different ethnic groups, including Western Europeans, the indigenous Khoisan, Bantu-speaking Africans, Indonesians and Malaysians were considered to be of Mixed Ancestry and were from Western Cape (n = 471). The study consisted of 550 diagnosed and histologically confirmed oesophageal squamous cell carcinoma or adenocarcinoma cases, who were recruited between 2000 and 2010 from Groote Schuur Hospital, Cape Town, Western Cape, South Africa. Cases were either from Black or Mixed Ancestry ethnic group. There was no restriction on recruitment criteria for age and gender of cases. Controls (n = 610) were healthy individuals without a previous history of cancer and were recruited from the same population groups and geographical area as the cases. Each participating subject was interviewed to collect information on demographic characteristics (age, gender, ethnicity), tobacco smoking and alcohol consumption and family history of cancer. Subjects with current or former smoking habits were classified as smokers. Alcohol consumers were defined as individuals who consumed more than 40 grams of alcohol per day. Family history of cancer was considered positive for individuals with at least one first-degree relative or two second-degree relatives having cancer. DNA was extracted from frozen blood samples using standard protocols. This study was approved by the University of Cape Town/Groote Schuur Hospital Human Ethics Research Commitee. Written informed consent was obtained from all participants recruited into the study.

### Selection of SNPs

Prior to genotyping, SNPs were selected from the HapMap database (Phase II+III release #28, August 10) based on their significantly different genotypic distributions between HapMap population of European ancestry (CEU; Utah residents with Northern and Western European ancestry from the CEPH collection) and 4 HapMap populations of African origin (ASW: African ancestry in Southwest USA; LWK: Luhya in Webuye, Kenya; MKK: Maasai in Kinyawa, Kenya; and/or YRI: Yoruba in Ibadan, Nigeria). We analysed 978 SNPs from 7 MMR genes and found 27 candidate polymorphisms in 5 MMR genes with significantly different genotypic distributions between African and non-African HapMap-populations. From those SNPs, ten were selected based on minor allele frequency (MAF >0.05) and their possible functional properties (e.g. nonsynonymous SNPs). In *MSH2* three SNPs were selected (rs17217772, Asn127Ser, c.380A>G; rs10188090, c.2635-765G>A; and rs3771280, c.1510+118T>C), three in *MSH3* (rs26279, Ala1045Thr, c.3133G>A; rs1428030, c.1341-12568A>G; and rs1805355, Pro231Pro, c.693G>A), two in *PMS1* (rs5742938, c. **−**21+639G>A; and rs13404927, c.699+3331G>A), one in *MLH1* (rs13320360, c.546-191T>C), and one in *MLH3* (rs28756991, Arg797His, c.2390G>A). No polymorphisms were selected in *MSH6* and *PMS2* genes, since genotypic distributions of polymorphisms were not significantly different between populations of African and non-African origin.

### Genotyping

All SNPs were analysed by allele-specific quantitative PCR assay [50], [51] using Roche LightCyler® 480II instrument. Two allele-specific primers, each specific for one of the two variants of the analysed SNP, and a common primer for each SNP were designed with WASP software [52]. To ensure better specificity, allele-specific primers contained an additional mismatch at penultimate position (second to last at 3’-end). Genotyping for each sample was performed in two parallel 3µL PCR reactions, one for each of the two alleles. Reactions contained 200 nM of one allele-specific primer, 200 nM of common primer, 5 ng of genomic DNA, and 1.5 µL KAPA™ SYBR® FAST qPCR Master Mix (2×) (Kapa Biosystems). Amplification conditions were as follows: initial denaturation for 3 min at 95°C; followed by 45 cycles of 5 sec at 95°C, 25 sec at 55–60°C (depending on the SNP), and 5 sec at 72°C; finally, melting curve analysis was performed. 10–20% of samples were re-genotyped to ascertain the reproducibility of the assay. Complete concordance between experiments was obtained. Primer sequences are available upon request.

### Statistical Analysis

Differences in demographic variables, lifestyle habits and genotypic frequencies between cases and control subjects were evaluated by using the Pearson’s Chi-Square (Χ^2^) test. Genotype data in control subjects from each ethnic group was checked for Hardy-Weinberg equlibrium using Fisher’s exact test. All genotypic analyses were performed assuming dominant and recessive models for the variant allele (i.e. minor allele in the control group) of each SNP. Crude odds ratios (ORs) and odds ratios adjusted for potential confounders (AORs), 95% confidence intervals (CIs) and *P-*values were obtained from logistic regression analysis using the SPSS (version 19) statistical package. For polymorphisms, the common homozygote genotype in the control subjects was set as the reference group. All reported values are two-sided, with *P-*value <0.05 considered as significant. Unadjusted significant *P-*values were corrected for multiple tests under the number of hypotheses tested (twenty per 10 SNPs in each ethnic group), using the Benjamini-Hochberg (BH) method [53]. Power of the study was calculated *post-hoc* using QUANTO (v1.2) [54].Haplotypes were constructed from our population genotype data (including missing genotypes) using PHASE (v2.1) software [55], [56]. Phasing of case and control haplotypes was performed separately. Samples with ≥90% certainty of phase estimates were considered in the analysis. In order to obtain reliable results, PHASE algorithm was applied 100 times for each haplotype using the -x option as instructed in the manual. The odds ratios (ORs) and their 95% confidence intervals (CIs) were estimated by Χ^2^ test. Significance for overall haplotype distribution between controls and cases was obtained with 1000 random permutations (P_1000_), where controls and cases were phased together.

Gene-gene interactions were explored using the model-based multifactor dimensionality reduction approach (MB-MDR) by applying a ‘mbmdr’ R-package to our whole dataset, including missing genotypes. General procedures of the three-step method and ‘mbmdr’ guidelines are fully described elsewhere [38], [57]. Briefly, in the first step of the algorithm, an association test between each multi-locus genotype and the phenotype is performed using logistic regression, where individuals with multi-locus genotype of interest are compared against the rest of the individuals (the latter are considered as the reference group in the analysis). Genotypes are then assigned into three categories: high-risk, low-risk and no-risk, accordingly. The second step of the algorithm explores association of pooled genotypes in low-risk and high-risk categories respectively, with the phenotype, using logistic regression analysis. Again, the rest of individuals are considered as the reference group. Significance of results is explored through Wald statistics in the third step. In this study, multi-order interaction with the most significant association between a specific multi-locus genotype and the phenotype, was considered the best model and was further adjusted for multiple testing by 1000 permutations approach (P_1000_). Stratification analysis for tobacco smoking and alcohol consumtion was performed using the SPSS package.

### *MSH3* and *PMS1* Expression Analysis

Freshly frozen normal and tumour oesophageal biopsies were obtained from 47 patients (Mixed Ancestry group) with histologically confirmed OSCC. Total RNA was extracted from homogenates of the tissue samples using RNeasy Mini Kit (Qiagen) according to manufacturer’s protocol. cDNA was prepared from 1 µg of total RNA using ImProm-II™ Reverse Transcription System (Promega) and was subsequently used as a template in quantitative PCR (qPCR). QPCR assays were performed with SYBR® FAST qPCR kit (KapaBiosystems) in 10 µL volume reactions containg 1 µL of cDNA and gene-specific primers for genes *GAPDH*, *MSH3* and *PMS1*, respectively. The following primer-pairs were used: GAPDH-fw (5′- GCC TGC TTC ACC ACC TTC) and GAPDH-rv (5′- GGC TCT CCA GAA CAT CAT CC); MSH3-fw (5′- GGC TCC TAT GTT CCT GCA GAA G) and MSH3-rv (5′- CCC TCT TCC TAG TTC ATC CAA GAT); PMS1-fw (5′- CCG TTA AGC ACA CCC AGT CAG) and PMS1-rv (5'- CAC AGG TTC AAT ATT CTC TCC CAC). All amplifications were performed as follows: initial denaturation at 95°C for 3 minutes, followed by 45 cycles at 95°C for 30 seconds, and 60°C for 30 seconds and 72°C for 10 seconds. Analysed genes in all 47 samples were amplified in triplicate using the Light Cycler 480II apparatus (Roche). *MSH3* and *PMS1* mRNA levels in each sample were normalized to *GAPDH* expression in the same sample using the efficiency corrected comparative *Ct* model:

?PCR efficiencies (E) were determined using LinRegPCR software [58]. Differences in expression levels between groups were evaluated with nonparametric Kurskal-Wallis test. Reported *P-*values were two-tailed.

### *In silico* Analysis of Amino Acid Substitutions

Predicting the putative effects of nonsynonymous SNPs on protein function was performed using SIFT (Sorting Intolerant from Tolerant) [59], [60], PolyPhen (Polymorphism Phenotype) [61], and Align-GVGD [62], [63] algorithms. From multiple protein sequence alignment, these bioinformatic tools provide prediction scores, indicating the probability that a SNP is tolerant or deleterious. SIFT predicts the functional importance of amino acid change based on sequence homology and physical properties of amino acids. Likewise, Align-GVGD combines the biophysical characteristics of amino acids and protein multiple sequence alignments, whereas PolyPhen predics the possible impact of an amino acid substitution using sequence conservation, phylogenetic and structural information characterizing the substitution. For all algorithms 26 MSH3 and 19 MLH3 protein sequences, were used as input sequences (Table S3). SIFT scores were designated as tolerant (0.201–1.00), borderline (0.101–0.20), potentially intolerant (0.051–0.10), or intolerant (0.00–0.05) [59], [60]. PolyPhen scores were classified as probably benign (0.000–0.999), borderline (1.000–1.249), potentially damaging (1.250–1.499), possibly damaging (1.500–1.999), or damaging (≥2.000) [61]. For Align-GVGD predictions, variants were classified according to AGVGD graded classifiers used with the software (http://agvgd.iarc.fr/agvgd_input.php).

## Supporting Information

Table S1 **Genotype distributions at each SNP in controls and oesophageal cancer cases in two ethnic groups of South African population.**
(PDF)

Table S2 **Individual SNP effects on OSCC risk in two ethnic groups of South African population.**
(PDF)

Table S3 **Computational analyses results.**
(PDF)
