# Supplementary material for: The Cumulative Effects of Polymorphisms in the DNA Mismatch Repair Genes and Tobacco Smoking in Oesophageal Cancer Risk
Source: PLoS One. 2012 May 18;7(5):e36962. doi: 10.1371/journal.pone.0036962 (PMC3356375; doi:10.1371/journal.pone.0036962)
Supplement: Table S1 — Genotype distributions at each SNP in controls and oesophageal cancer cases in two ethnic groups of South African population. (PDF) [file pone.0036962.s001.pdf]

Table S1: Genotype distributions at each SNP in controls and oesophageal cancer cases in two ethnic groups of South African population

Characteristics	Black group		Mixed Ancestry group	
	**Controls (%)**	**Cases (%)**	**Controls (%)**	**Cases (%)**
MSH2, rs17217772				
AA	301 (87.7)	302 (88.6)	250 (94.7)	191 (93.6)
AG	41 (12.0)	37 (10.8)	14 (5.3)	12 (5.9)
GG	1 (0.3)	2 (0.6)	0 (0.0)	1 (0.5)
Total	343	341	264	204
Minor allele (frequency)	G (0.06)	G (0.06)	G (0.03)	G (0.03)
MSH2, rs10188090				
AA	279 (81.6)	284 (82.8)	119 (44.9)	102 (49.8)
AG	60 (17.5)	54 (15.7)	117 (44.2)	78 (38.0)
GG	3 (0.9)	5 (1.5)	29 (10.9)	25 (12.2)
Total	342	343	265	205
Minor allele (frequency)	G (0.10)	G (0.09)	G (0.33)	G (0.31)
MSH2, rs3771280				
CC	261 (77.0)	272 (79.1)	111 (41.7)	92 (45.5)
CT	74 (21.8)	65 (18.9)	111 (41.7)	80 (39.6)
TT	4 (1.2)	7 (2.0)	44 (16.5)	30 (14.9)
Total	339	344	266	202
Minor allele (frequency)	T (0.12)	T (0.11)	T (0.37)	T (0.35)
MSH3, rs26279				
AA	113 (32.9)	122 (35.8)	117 (44.5)	82 (40.2)
AG	169 (49.1)	167 (49.0)	126 (47.9)	89 (43.6)
GG	62 (18.0)	52 (15.2)	20 (7.6)	33 (16.2)
Total	344	341	263	204
Minor allele (frequency)	G (0.43)	G (0.40)	G (0.32)	G (0.38)
MSH3, rs1428030				
AA	188 (55.0)	167 (48.8)	168 (63.6)	118 (58.7)
AG	125 (36.5)	149 (43.6)	86 (32.6)	72 (35.8)
GG	29 (8.5)	26 (7.6)	10 (3.8)	11 (5.5)
Total	342	342	264	201
Minor allele (frequency)	G (0.27)	G (0.29)	G (0.20)	G (0.23)
MSH3, rs1805355				
GG	177 (52.2)	172 (50.1)	160 (60.4)	116 (57.1)
GA	130 (38.3)	147 (42.9)	92 (34.7)	77 (37.9)
AA	32 (9.4)	24 (7.0)	13 (4.9)	10 (4.9)
Total	339	343	265	203
Minor allele (frequency)	A (0.29)	A (0.28)	A (0.22)	A (0.24)
PMS1, rs5742938				
GG	244 (70.9)	232 (67.2)	67 (25.2)	78 (38.4)
AG	94 (27.3)	104 (30.1)	122 (45.9)	76 (37.4)
AA	6 (1.7)	9 (2.6)	77 (28.9)	49 (24.1)
Total	344	345	266	203
Minor allele (frequency)	A (0.15)	A (0.18)	G (0.48)	G (0.43)
PMS1, rs13404927				
GG	223 (65.8)	231 (67.5)	207 (78.4)	152 (74.5)
GA	100 (29.5)	101 (29.5)	51 (19.3)	46 (22.6)
AA	16 (4.7)	10 (2.9)	6 (2.3)	6 (2.9)

Total	339	342	264	204
Minor allele (frequency)	A (0.19)	A (0.18)	A (0.12)	A (0.14)
MLH1, rs13320360				
TT	232 (68.2)	244 (71.1)	233 (88.2)	177 (87.2)
TC	98 (28.8)	93 (27.1)	30 (11.4)	23 (11.3)
CC	10 (2.9)	6 (1.7)	1 (0.4)	3 (1.5)
Total	340	343	264	203
Minor allele (frequency)	C (0.17)	C (0.15)	C (0.06)	C (0.07)
MLH3, rs28756991				
GG	267 (78.1)	273 (79.1)	244 (92.4)	171 (83.4)
GA	70 (20.5)	71 (20.6)	20 (7.6)	30 (14.6)
AA	5 (1.4)	1 (0.3)	0 (0.0)	4 (2.0)
Total	342	345	264	205
Minor allele (frequency)	A (0.12)	A (0.11)	A (0.04)	A (0.09)
