# Supplementary material for: The Cumulative Effects of Polymorphisms in the DNA Mismatch Repair Genes and Tobacco Smoking in Oesophageal Cancer Risk
Source: PLoS One. 2012 May 18;7(5):e36962. doi: 10.1371/journal.pone.0036962 (PMC3356375; doi:10.1371/journal.pone.0036962)
Supplement: Table S2 — Individual SNP effects on OSCC risk in two ethnic groups of South African population. (PDF) [file pone.0036962.s002.pdf]

Table S2: Individual SNP effects on OSCC risk in two ethnic groups of South African population

SNP<br>Genetic model	Black Ancestry group<br>AOR (95% CI)	*P*-value	Mixed Ancestry group<br>AOR (95% CI)	*P*-value
rs17217772, Asn127Ser				
GG vs AA/AG	1.52 (0.11-20.37)	0.753	ND	
GG/AG vs AA	1.11 (0.66-1.88)	0.687	1.65 (0.65-4.15)	0.292
rs10188090, c.2635-765G>A				
GG vs AA/AG	1.27 (0.26-6.29)	0.768	1.19 (0.56-2.55)	0.644
GG/AG vs AA	0.98 (0.63-1.53)	0.921	0.86 (0.55-1.36)	0.519
rs3771280, c.1510+118T>C				
TT vs CC/CT	1.63 (0.41-6.54)	0.489	0.84 (0.43-1.62)	0.593
TT/CT vs CC	1.01 (0.67-1.53)	0.956	0.82 (0.52-1.30)	0.401
rs26279, Ala1045Thr				
GG vs AA/AG	0.88 (0.56-1.38)	0.569	**2.78 (1.34-5.78)**	**0.006**
GG/AG vs AA	1.05 (0.73-1.50)	0.808	1.35 (0.84-2.16)	0.219
rs1428030, c.1341-12568A>G				
GG vs AA/AG	0.93 (0.50-1.71)	0.808	1.48 (0.52-4.22)	0.462
GG/AG vs AA	1.31 (0.93-1.84)	0.118	1.13 (0.71-1.81)	0.608
rs1805355, Pro231Pro				
AA vs GG/GA	0.67 (0.36-1.24)	0.206	1.19 (0.43-3.32)	0.740
AA/GA vs GG	1.10 (0.78-1.54)	0.583	1.06 (0.67-1.70)	0.794
rs5742938, c.-21+639G>A				
AA vs GG/GA; (GG vs AA/AG)[a]	1.89 (0.54-6.57)	0.319	**1.92 (1.17-3.15)**	**0.010**
AA/GA vs GG; (GG/AG vs AA)[a]	0.99 (0.68-1.43)	0.946	1.41 (0.83-2.37)	0.201
rs13404927, c.699+3331G>A				
AA vs GG/GA	0.74 (0.31-1.75)	0.488	1.00 (0.24-4.20)	0.998
AA/GA vs GG	1.02 (0.71-1.47)	0.911	1.19 (0.65-1.93)	0.685
rs13320360, c.546-191T>C				
CC vs TT/CT	0.61 (0.20-1.87)	0.387	8.28 (0.53-129.1)	0.131
CC/CT vs TT	0.84 (0.58-1.22)	0.358	1.03 (0.51-2.11)	0.926
rs28756991, Arg797His				
AA vs GG/GA	0.17 (0.02-1.46)	0.106	ND	
AA/GA vs GG	0.93 (0.62-1.40)	0.735	**2.09 (1.03-4.24)**	**0.042**

[a]Minor alleles are different between the two ethnic groups, hence genetic model indicated in brackets was investigated in Mixed Ancestry group. Significant associations are printed in bold. AOR, odds ratio adjusted for age, gender,smoking status, alcohol intake, place of birth and family history of cancer; CI, confidence interval; ND, not determined (zero genotypes were found in one genotype group).
