# Supplementary material for: The Cumulative Effects of Polymorphisms in the DNA Mismatch Repair Genes and Tobacco Smoking in Oesophageal Cancer Risk
Source: PLoS One. 2012 May 18;7(5):e36962. doi: 10.1371/journal.pone.0036962 (PMC3356375; doi:10.1371/journal.pone.0036962)
Supplement: Table S3 — Computational analyses results. (PDF) [file pone.0036962.s003.pdf]

Table S3: Computational analyses results

dbSNP ID	AA Change	Gene	SIFT	PolyPhen	Align-GVGD
rs26279	Ala1045Thr	*MSH3*	0.57 (neutral)	0.380 (neutral)	208.40/31.45 (neutral)
rs28756991	Arg797His	*MLH3*	**0.19 (borderline)**	**2.380 (damaging)**	353.86/0.00 (neutral)

For all algorithms 26 MSH3 and 19 MLH3 protein sequences, were used as input sequences Full 26 MSH3 sequences were identified for the following species: *Homo sapiens* (P20585), *Dictyostelium discoideum* (Q1ZXH0), *Aspergillus niger* (A2R1F6), *Scheffersomyces stipitis* (A3LU10), *Schizosaccharomyces pombe* (P26359), *Mus musculus* (P13705), *Arabidopsis thaliana* (O65607), *Yarrowia lipolytica* (Q6CHE5*),* *Phaeosphaeria nodorum* (Q0UXL8), *Chaetomium globosum* (Q2HFD4), *Cryptococcus neoformans* (P0CO93), *Emericella nidulans* (Q5B6T1), *Botryotinia fuckeliana* (A6RPB6), *Coccidioides immitis* (Q1DQ73), *Kluyveromyces lactis* (Q6CSR1), *Meyerozyma guilliermondii* (A5DEV6), *Lodderomyces elongisporus* (A5DYV8), *Candida albicans* (Q59Y41), *Magnaporthe oryzae* (A4R0R0), *Ajellomyces capsulata* (A6R7S1), *Sclerotinia sclerotiorum* (A7EC69), *Neosartorya fischeri* (A1DCB2), *Ashbya gossypii* (Q759V4), *Vanderwaltozyma polyspora* (A7TTQ1), *Debaryomyces hansenii* (Q6BW83) and *Saccharomyces cerevisiae* (A6ZTR3); whereas MLH3 sequences were identified for the following 19 species: *Homo sapiens* (Q9UHC1), *Saccharomyces cerevisiae* (Q12083), *Dictyostelium discoideum* (Q54QI0), *Rattus norvegicus* (D3ZGD6), *Ectocarpus siliculosus* (D7G9D8), *Arabidopsis thaliana* (F4JN26), *Sordaria macrospora* (D1ZLU0), *Monodelphis domestica* (F7GHH3), *Sus scrofa* (F1S2R6), *Gallus gallus* (E1C7F3), *Xenopus tropicalis* (F6YUY9), *Xenopus laevis* (Q6GNZ4), *Bos taurus* (E1BNK2), *Canis familiaris* (E2RD40), *Callithrix jacchus* F7IL84), *Macaca mulatta* (F7HAZ3), *Penicillium marneffei* (B6Q260), *Neosartorya fischeri* (A1CXX4) and *Aspergillus clavatus* (A1CGV6).
